# 

**Published:** 1884-03

**Authors:** 


### THE BRISTOL

### Medico-Chirurgical Journal.

A JOURNAL OF THE MEDICAL SCIENCES FOR THE
WEST OF ENGLAND AND SOUTH WALES.

*PUBLISHED UNDER THE AUSPICES OF*
*THE BRISTOL MEDICO-CHIRURGICAL SOCIETY.*

EDITED BY
J. GREIG SMITH,
M.A., F.R.S.E.

"Scire est nescire, nisi id me
Scire alius sciret."

SURGEON GEN'L'S OFFICE
LIBRARY.
97425

*VOL. II.*

BRISTOL: J. W. ARROWSMITH;
LONDON: J. & A. CHURCHILL, 11 NEW BURLINGTON STREET.
1884.

WI
BR323

J.W. ARROWSMITH,

Printer and

Publisher,

QUAY STREET, BRISTOL.

### CONTENTS OF VOL. II.

ORIGINAL PAPERS :— *Page.*

On Flat-foot, and its Cure by Operation. By ALEXANDER OGSTON, C.M. ... ... ... ... ... ... ... ... ... ... 1

The Effects of certain Anatomical Relations. By JAMES CANTLIE, M.A., M.B., F.R.C.S. ... ... ... ... ... ... 21

On giving Rest to the Digestive Organs by the Use of Peptonised Food. By W. H. SPENCER, M.A., M.D. Cantab. ... ... ... ... ... ... ... ... ... ... ... 32

The Nature and Treatment of Chorea. By E. LONG FOX, M.D., F.R.C.P. ... ... ... ... ... ... ... ... ... ... 73

Ingrowing Toe-nail. By THE EDITOR ... ... ... ... ... ... ... 99

Glaucoma. By F. R. CROSS, M.B. Lond., F.R.C.S. ... ... 145

An Undescribed Form of Stricture at the Orifice of the Male Urethra. By THE EDITOR ... ... ... ... ... ... ... 154

A New Neurosis (Thomsen's Disease). By WILLIAM H. SPENCER, M.A., M.D. Cantab. ... ... ... ... ... ... 159

The Etiology of Catarrh. Inaugural Address by GEORGE F. BURDER, M.D., F.R.C.P. ... ... ... ... ... ... ... ... 217

Some Points in the Pathology of General Paralysis of the Insane. By T. STEELE SHELDON, M.B. Lond. ... ... 235

On the Diagnosis of Abscess of the Liver. By WILLIAM JOHNSTONE FYFFE, M.D. ... ... ... ... ... ... ... 245

CLINICAL RECORDS :—

Two Cases of Gastric Ulcer treated by Peptonised Enemata. By Drs. SPENCER and SHAW ... ... ... ... ... ... 41

Case of Spontaneons Cure of Spina Bifida, followed by Hydrocephalus. By E. LONG FOX, M.D., F.R.C.P. ... 45

Case of Rupture of the Right Auricle of the Heart. By GEORGE THOMPSON, M.D., L.R.C.P. Lond. ... ... ... 48

Page.

Case of Successful Immediate Suturing of the Olecranon for Simple Fracture. By W. P. KEALL, M.R.C.S. ... ... 50

Case of Death from Fat Embolism after Bone-setting. By Arthur W. PRICHARD, M.R.C.S. ... ... ... ... ... 54

Case of Diphtheria treated by Tracheotomy, Peptonised Enemata, and Iodoform. By R. SHINGLETON SMITH, M.D., M.R.C.P. ... ... ... ... ... ... ... ... ... 61

Short Reports of Twelve Cases of Ligature of some of the Main Arteries occurring during the past few months at the Bristol Royal Infirmary. By J. PAUL BUSH ... ... 110

Case of Cerebral Abscess following Slight Injury. By A. N. GODBY GIBBS ... ... ... ... ... ... ... ... ... ... 121

Case of Malignant Pustule. By LIONEL A. WEATHERLY, M.D. 124

Case of Tumour (Columnar Papilloma) in Fourth Ventricle of Brain, with Hydrocephalus. By J. TAYLOR, M.R.C.S. 128

Three Cases of Intestinal Obstruction. By ALFRED SHEEN, M.D. 167

Case of Subacute Poliomyelitis Anterior, with Commencing Phthisis. Treatment by Ergotin and Iodoform. Recovery. By R. SHINGLETON SMITH, M.D., M.R.C.P. ... ... ... 174

Sudden Death from Asphyxia in a Phthisical Boy. By W. BARRETT ROUÉ, M.D., M.S., F.R.A.S. ... ... ... 181

Case of Subdiaphragmatic Abscess of the Left Side. By J. HARPER, L.R.C.P. Lond. ... ... ... ... ... ... ... ... 184

Notes of Cases of Diphtheritic Paralysis, with Remarks. By ANDREW S. CURRIE, M.D. ... ... ... ... ... ... ... 186

A Case of Laryngeal Phthisis, in which the Windpipe was opened on account of Dyspnœa. By CHRISTOPHER ELLIOTT, M.D. ... ... ... ... ... ... ... ... ... ... 260

Note on the After Treatment of Scarlet Fever by Scalded Oatmeal. By GEORGE SMITH, M.R.C.S.... ... ... ... 263

Two Cases of Hæmophilia. By THE EDITOR ... ... ... ... 264

Lateral Sclerosis of the Spinal Cord. By J. E. SHAW, M.B. 270

NOTES ON BOOKS ... ... ... ... ... ... ... ... ... 63, 132, 196, 275

MEDICAL EXTRACTS ... ... ... ... ... ... ... ... 70, 136, 203, 280

### LIST OF SUBSCRIBERS

TO THE

### Bristol Medico-Chirurgical Journal.

*Those marked * are Members of the Society.*

| *Name.* | *Residence.* |
|---|---|
| Ackland, W. H., M.D. St. And. ... ... ... | Bideford. |
| Adams, George ... ... ... ... ... ... ... | 5 Oakfield Road, Clifton. |
| Adams, James, M.D. Aberd. ... ... ... ... | Ashburton. |
| *Adams, John Barfield... ... ... ... ... ... | 3 Selwood Villas, Bristol. |
| Alexander, James, M.D., M.Ch. Dub. ... ... | Bishop's Place, Paignton. |
| Alford, George Ernest ... ... ... ... ... ... | 5 Royal Crescent, Weston-super-Mare. |
| Alford, Henry J., M.D. Lond. ... ... ... | 20 The Crescent, Taunton. |
| Alford, Richard ... ... ... ... ... ... ... | 6 Oriel Terrace, Weston-super-Mare. |
| *Andrews, Onslow, M.D. St. And.... ... ... | 159 White Ladies Road, Bristol. |
| Anstie, Thomas B. ... ... ... ... ... ... | 21 Northgate Street, Devizes. |
| *Atchley, George F., M.B. Lond.... ... ... | Tyndall's Park, Bristol. |
| Baretti, T. G. L'Enardi ... ... ... ... ... | 49 Royal York Crescent, Clifton. |
| *Baron, B. J., M.B., C.M. Ed. ... ... ... ... | 12 Richmond Hill, Clifton. |
| Batten, Rayner W., M.D. Lond. ... ... ... | 1 Brunswick Square, Gloucester. |
| *Beddoe, John, M.D. Ed., B.A. Lond., F.R.S. | Mortimer House, Clifton. |
| Bennett, W. S. ... ... ... ... ... ... ... ... | 12 The Terrace, Penzance. |
| Berry, F. C., M.D., B.Ch. Dub. ... ... ... | Lynton. |
| *Blacker, A. E. ... ... ... ... ... ... ... ... | 7 Unity Street, Bristol. |
| Blacker, E. ... ... ... ... ... ... ... ... ... | Midsomer Norton. |
| Blaxall, F. H., M.D. St. And. ... ... ... | Clan Lodge, Bath. |
| Blomfield, Arthur G., M.D. Aberd. ... ... | Devon and Exeter Hospital, Exeter. |
| *Board, Edmund C. ... ... ... ... ... ... ... | 7 Caledonia Place, Clifton. |
| Bond, Francis T., M.D. Lond. ... ... ... | 1 Beaufort Buildings, Gloucester. |
| Bousfield, E. C. ... ... ... ... ... ... ... ... | 363 Old Kent Road, London. |
| Bower, Ernest D. ... ... ... ... ... ... ... | 1 Norfolk Terrace, Gloucester. |
| Boyle, Thomas ... ... ... ... ... ... ... ... | Newquay. |
| *Boys, Arthur Henry ... ... ... ... ... ... | Lodway villa, Pill. |
| Bridgman, I. T. ... ... ... ... ... ... ... ... | Berkeley. |
| Broom, John, M.D. Brussels ... ... ... ... | St. Paul's Road, Clifton. |
| Broster, Alfred, M.D. Giessen ... ... ... | Zetland Road, Bristol. |
| Brown, G. Arthur ... ... ... ... ... ... ... | Tredegar. |
| *Burder, George F., M.D. Aberd.... ... ... | 63 Oakfield Road, Clifton. |
| Burges, R. E., M.D., M.Ch., B.A. Qu. Univ. I. | Kettering, Northamptonshire. |
| Burroughs, E. F. H. ... ... ... ... ... ... | Stackpole Road, Bristol. |
| Bush, J. Paul ... ... ... ... ... ... ... ... ... | Royal Infirmary, Bristol. |
| Cann, Francis M. ... ... ... ... ... ... ... ... | Sefton House, Dawlish. |
| Carey, J., M.D. Lond.... ... ... ... ... ... ... | 2 Hamdon Villas, Taunton. |
| *Carr, Alexander ... ... ... ... ... ... ... ... | Wells Road, Bristol. |
| *Carter, W. F.... ... ... ... ... ... ... ... ... | Coronation Road, Bristol. |
| *Challacombe, J. P., M.D. Ed. ... ... ... | 143 Cheltenham Road, Bristol. |
| Clark, Thomas ... ... ... ... ... ... ... ... | Dunster. |
| *Clark, T. E., M.D., C.M. Aberd. ... ... ... | 3 Victoria Square, Clifton. |
| Clarke, Fred. H. ... ... ... ... ... ... ... ... | Chillington, near Kingsbridge. |
| *Clarke, W. Michell ... ... ... ... ... ... ... | 2 York Buildings, Clifton. |
| Clay, R. Hogarth, M.D. Ed. ... ... ... ... | 4 Windsor Villas, Plymouth. |
| Coathupe, Edwin W. ... ... ... ... ... ... | 7 Clifton Park, Clifton. |
| *Coe, R. W. ... ... ... ... ... ... ... ... ... | 14 Berkeley Square, Bristol. |

| | |
|---|---|
| Coker, T. V. | 2 Chesterfield Place, Clifton. |
| Cole, Thomas, M.D. Lond. | 17 Paragon, Bath. |
| *Collins, H. W. | Wrington. |
| Colman, Thomas J., M.D. Ed. | Redland Road, Bristol. |
| Colmer, Ptolemy S. H., M.D. Dur. | South Street House, Yeovil. |
| Colthurst, J. B. | Wincanton. |
| *Connolly, William, M.D. St. And. | 3 Goldney Road, Clifton. |
| Cooke, A. S. | Badbrook House, Stroud. |
| Cooper, Herbert | Rosslyn Hill, Hampstead, London, N.W. |
| *Corbould, George G. | 22 Berkeley Square, Bristol. |
| Cornwall, James | Fairford. |
| Couch, James | Christina Street, Swansea. |
| Cripps, Edward | Cirencester. |
| *Crisp, Nathaniel | Chandos House, Keynsham. |
| *Cross, F. Richardson, M.B. Lond. | Chandos Villa, Clifton. |
| *Crossman, Edward | Hambrook. |
| Cunningham, A. G., M.B., C.M. Aberd. | Stapleton Road, Bristol. |
| Currie, Andrew S., M.D. Ed. | Oakfield Villa, Lydney. |
| Daly, George H., M.D. St. And. | Burley House, Chippenham. |
| *Davey, James G., M.D. St. And. | 4 Redland Park, Bristol. |
| *Davies, D. S., M.B. Lond. | 41 Queen Square, Bristol. |
| Davis, Theodore, M.D. Lond. | Beachcroft, Clevedon. |
| Day, Percy Howard | Haverigg, Millom, Carnforth. |
| Dening, Edwin | Stow-on-the-Wold. |
| *Dew, Henry R. | 25 Berkeley Square, Bristol. |
| *Dobson, Nelson C. | 27 Victoria Square, Clifton. |
| *Dowson, C. H. | Tyndall's Park, Bristol. |
| Doyne, R. W. | Fishponds. |
| Dunbar, Eliza L. Walker, M.D. Zürich | 9 Oakfield Road, Clifton. |
| Eadon, W. F. Bailey | Hambrook. |
| *Eager, Reginald, M.D. Lond. | Northwoods. |
| Edgeworth, T. F. | 1 Stokes Croft, Bristol. |
| *Elliott, Christopher, M.D. Dub. | 155 White Ladies Road, Bristol. |
| *Elwin, J. Fountain | 17 Clyde Road, Bristol. |
| *Evans, J. Fenton, M.B., C.M. Ed. | Royal Infirmary, Bristol. |
| *Ewens, John | 20 Cotham New Road, Bristol. |
| Fairbanks, W., M.D., C.M. Ed. | Wells. |
| Fasulo, Violetta | Nurses' Institute, Bournemouth. |
| *Fendick, R. George | 3 Claremont Place, Clifton. |
| Fernie, Andrew, M.D. Dur. | Barnstaple. |
| Fernie, James | Stratton St. Margaret. |
| Ferris and Co. | Union Street, Bristol. |
| Fiddian, Alexander P., M.B. Lond. | 6 Brighton Terrace, Cardiff. |
| Fletcher, Henry Studd | Lydbrook. |
| Forty, D. H. | Wotton-under-Edge. |
| Fox, Arthur E. W., M.B., C.M. Ed. | 16 Gay Street, Bath. |
| *Fox, Bonville B., M.B., M.A. Oxon. | Brislington. |
| *Fox, E. Long, M.D. Oxon. | Church House, Clifton. |
| Fraser, Græme B. | 7 Lower Church Road, Weston-s.-Mare. |
| Fry, J. Farrant | 203 St. Helen's Road, Swansea. |
| Fry, John B. | Swindon. |
| Fuller, J. | Long Ashton. |
| *Fyffe, W. Johnstone, M.D. Dub. | 2 Rodney Place, Clifton. |
| Gardiner, George | 1 Redcliff Hill, Bristol. |
| *Gibbs, Alfred N. Godby | Children's Hospital, Bristol. |

| | |
|---|---|
| Giles, G. F., M.D. | Lower Redland Road, Bristol. |
| *Gloag, G. A. | 6 College Green, Bristol. |
| Gooding, J. Callender, M.D. Ed. | Berkeley Street, Cheltenham. |
| Goodridge, Henry F. A., M.D. Lond. | 10 Brook Street, Bath. |
| Goss, T. Biddulph | 36 Paragon, Bath. |
| Gowing, B. C. | Avon House, Salisbury. |
| Grace, Alfred | Chipping Sodbury. |
| Grace, Edward Mills | Park House, Thornbury. |
| Grace, Henry | Kingswood. |
| Grace, W. G. | 57 Stapleton Road, Bristol. |
| Green, F. K. | 3 Gay Street, Bath. |
| Gregory, George S. | The Green, Stroud. |
| *Griffiths, L. M. | 9 Gordon Road, Clifton. |
| Grote, G. W. | 32 Portland Square, Bristol. |
| Harper, Joseph | Bear Street, Barnstaple. |
| *Harrison, A. J., M.B. Lond. | Guthrie Road, Clifton. |
| *Harsant, W. H. | 16 Pembroke Road, Clifton. |
| Hawkins, Cæsar F. | 7 Berkeley Square, Bristol. |
| Hayman, A. G. | 1 Pembroke Road, Clifton. |
| *Heaven, John C. | Avonmouth. |
| *Herapath, C. K. C. | 11 Brunswick Square, Bristol. |
| *Hetling, H. E. | Stapleton Road, Bristol. |
| Hichens, James S. | 17 Green Lane, Redruth. |
| Hine, S. D. | Ilminster. |
| Hinton, Joseph | Warminster. |
| Hopkins, H. Culliford | 4 Gay Street, Bath. |
| Howse, William | London Street, New Swindon. |
| Howsin, E. Arthur, M.D. St. And. | Stroud. |
| Hyatt, Brownlow N. | Park View, Shepton Mallet. |
| *Imlay, G. A., M.B., C.M. Aberd. | 4 Apsley Villas, Bristol. |
| Infirmary, The Royal | Bristol. |
| Institution, Liverpool Medical (*Librarian*, William Jones.) | |
| *Isaac, G. Washington M.B., C.M. Ed. | Manor House, Brixton Rise, Lond., S.W. |
| James, J. Bowen | Woodlands, Aberaman, Aberdare. |
| Jenkins, John Henry | Liskeard. |
| Jessop, Charles Moore | The Willows, Fulwood Park, Preston. |
| Jones, Evan | Ty-mawr, Aberdare. |
| Joynes, Francis James | Eagle House, Dursley. |
| *Keall, W. P. | Coronation Road, Bristol. |
| Kempe, Arthur W. | 20 St. Sidwell's, Exeter. |
| Kesteven, W. B., M.D. St. And. | Little Park, Enfield, Middlesex. |
| Kinneir, R. | The Tower House, Malmesbury. |
| Kirkman, J. Miller | Wootton Bassett. |
| Laing, W. A. Gordon, M.B., C.M. Glasg. | Mevagissey. |
| *Lansdown, F. Poole | 19 White Ladies Road, Clifton. |
| Latimer, H. A. | 72 Mansel Terrace, Swansea. |
| *Lawrence, A. E. Aust, M.D., C.M. Aberd. | 15 Richmond Hill, Clifton. |
| Lawrence, A. G., M.D. St. And. | Chepstow. |
| *Lawrence, Henry | 9 Park Row, Bristol. |
| *Laxton, Henry | 12 Apsley Road, Clifton. |
| *Lewis, J. R., M.B., C.M. Glasg. | 20 Gloucester Road, Bristol. |
| Liddon, William, M.B. Lond. | Church Square, Taunton. |
| Lloyd, Walter E. | 60 York Road, Bristol. |
| Lodge, John | Keynsham. |
| Logan, Frederic T. B. | Dean Lane, Bristol. |

| | |
|---|---|
| LOVELL, W. DAY ... ... ... ... ... ... ... | The Abbey, Bradford-on-Avon. |
| LOVELL, WILLIAM FREDERICK ... ... ... ... | West End House, Compton Martin. |
| LOWE, T. PAGAN ... ... ... ... ... ... ... | Royal United Hospital, Bath. |
| *LUFFMAN, W. C. ... ... ... ... ... ... ... | 8 Cotham New Road, Bristol. |
| *LYONS, A. DE COURCY, M.B., C.M. Aberd. ... | Henley Lodge, Yatton. |
| MACLEAN, J. CAMPBELL, M.B., C.M. Ed. ... | Swindon House, Swindon. |
| MAGRATH, J. A., M.D. Glasg. ... ... ... ... | 7 Den Crescent, Teignmouth. |
| MANNING, H. J. ... ... ... ... ... ... ... | Laverstock House, Salisbury. |
| *MARCHANT, W. R. F., M.D. St. And. ... ... | 91 White Ladies Road, Bristol. |
| MARLEY, HENRY F. ... ... ... ... ... ... | The Nook, Padstow. |
| MARSH, CHARLES J. ... ... ... ... ... ... | Yeovil. |
| *MARSHALL, HENRY, M.D. Ed., F.R.S.E. ... | 28 Caledonia Place, Clifton. |
| MASON, FREDERICK ... ... ... ... ... ... | 20 Belmont, Bath. |
| MASON, S. B. ... ... ... ... ... ... ... ... | 12 Park Terrace, Pontypool. |
| MASON, WILLIAM ... ... ... ... ... ... ... | St. Austell. |
| MATTHEWS, CHARLES E. ... ... ... ... ... | Stapleton Road, Bristol. |
| MAYOR, T. O. ... ... ... ... ... ... ... ... | 40 Apsley Road, Clifton. |
| *MEAD, H. RIMMINGTON ... ... ... ... ... | Fishponds. |
| MEREDITH, JOHN, M.D. Ed. ... ... ... ... | Wellington. |
| MILNE, JOHN, M.B., C.M. Aberd. ... ... ... | 97 Clarence Road, Bristol. |
| MILWARD, JAMES ... ... ... ... ... ... ... | 54 Charles Street, Cardiff. |
| MOORHEAD, J., M.D., M.A. Qu. Univ. I. ... | 1 Royal Terrace, Weymouth. |
| MORGAN, E. RICE ... ... ... ... ... ... ... | Morriston, Swansea. |
| MORGAN, JOHN ... ... ... ... ... ... ... ... | Langport. |
| MORGAN, WILLIAM, M.D. Aberd. ... ... ... | Dumfries Place, Cardiff. |
| MOYNAN, W. ARTHUR, M.D., M.Ch. Qu. Univ. I. | Somerset County Asylum, Wells |
| MURRAY, WILLIAM, M.B., C.M. Ed. ... ... | Staple Hill. |
| NAPIER, T. W. A., M.B., C.M. Aberd. ... ... | Strathwye, Tintern. |
| NEEDHAM, FREDERICK, M.D. St. And. ... ... | Barnwood House, Gloucester. |
| *NEWMAN, CHARLES ... ... ... ... ... ... | 19 Portland Square, Bristol. |
| NEWTON, CHARLES JOHN ... ... ... ... ... | Oriel Lodge, Cheltenham. |
| NICHOLSON, T. D., M.D., C.M. Ed. ... ... | 2 White Ladies Road, Clifton. |
| NORMAN, J. H. ... ... ... ... ... ... ... ... | Goodleigh, Winkleigh. |
| NORMAN, J. W. ... ... ... ... ... ... ... ... | Edde Cross House, Ross. |
| *NORTON, J. A., M.D., C.M. Aberd. ... ... ... | 8 Redcliff Hill, Bristol. |
| *NORTON, T. CHALMERS ... ... ... ... ... | 12 King Square, Bristol. |
| NOURSE, W. J. CHICHELE ... ... ... ... ... | Morchard Bishop. |
| OLLERHEAD, T. J. ... ... ... ... ... ... ... | Hillbury, Minehead. |
| *ORMEROD, HENRY ... ... ... ... ... ... ... | Westbury-upon-Trym. |
| PADLEY, GEORGE ... ... ... ... ... ... ... | 2 Northampton Terrace, Swansea. |
| PAINE, WILLIAM HENRY, M.D. Aberd. ... ... | Stroud. |
| *PARETTE, JAMES ... ... ... ... ... ... ... | 7 College Green, Bristol. |
| PARRY, J. H. ... ... ... ... ... ... ... ... | 99 Ashley Road, Bristol. |
| *PARSON, T. COOKE ... ... ... ... ... ... ... | 21 White Ladies Road, Bristol. |
| PARSONS, FRANCIS J. C. ... ... ... ... ... | King Square, Bridgwater. |
| PARSONS, J. D. F., M.D. St. And. ... ... ... | 13 Dowry Square, Clifton. |
| PARTRIDGE, THOMAS ... ... ... ... ... ... | Stroud. |
| PAULI, G. C. P. ... ... ... ... ... ... ... | 240 York Road, Bristol. |
| PEARCE, EDWARD T. ... ... ... ... ... ... | Holsworthy. |
| PEARSE, FRED. E., M.D. St. And. ... ... ... | Hall House, Frome. |
| *PENNY, W. J. ... ... ... ... ... ... ... ... | General Hospital, Bristol. |
| PENRUDDOCKE, CHARLES ... ... ... ... ... | Winchcombe. |
| PERKINS, E. B. STEELE ... ... ... ... ... | 21 Magdalen Street, Exeter. |
| PERKINS —., M.D. New York ... ... ... ... | 14 Victoria Square, Clifton. |
| PERRIN, J. D. ... ... ... ... ... ... ... ... | Temple Cloud. |

| | |
|---|---|
| Phelps, W. Harford G., M.D. Aberd. | Weston-super-Mare. |
| Phelps, William H. | 123 Ashley Road, Bristol. |
| Philipps, S. Rees, M.D., M.Ch. Qu. Univ. I. | Wonford House, Exeter. |
| Phillips, Edward E. | Broadwater House, Southend. |
| Philpots, Edward P., M.D., C.M. Aberd. | Bourne Hall, Bournemouth. |
| *Pickering, C. F. | 12 Berkeley Square, Bristol. |
| Pizey, G. F. P. | Rydal Villa, Clevedon. |
| Porter, P. | 109 Fore Street, Saltash. |
| Potter, Samuel R., M.B., B.A. Dub. | Collumpton. |
| *Pountney, W. E., M.D., C.M. Ed. | 107 White Ladies Road, Bristol. |
| Powell, William, M.B. Lond. | Hill Garden, Torquay. |
| Prankerd, John | Langport. |
| *Prichard, Arthur W. | 31 St. Paul's Road, Clifton. |
| *Prichard, Augustin, M.D. Berlin | 4 Chesterfield Place, Clifton. |
| *Prichard, J. E., M.B., B.A. Oxon. | 243 Cheltenham Road, Bristol. |
| Prideaux, T. Engledue P. | Wellington. |
| *Prowse, Arthur B., M.D. Lond. | 4 Rodney Cottages, Clifton. |
| Randolph, Charles | Milverton. |
| Rawlings, John Adams | 4 Northampton Terrace, Swansea. |
| Redwood, T. Hall, M.D., C.M., M.A. Dur. | The Lawn, Rhymney. |
| Rogers, George, M.D. St. And. | 6 Portland Square, Bristol. |
| Rossiter, George F., M.B. Lond. | Cairo Lodge, Weston-super-Mare. |
| *Roué, W. Barrett, M.D., M.S. Dur. | 2 Buckingham Place, Clifton. |
| Roxburgh, Robert, M.B. Ed. | 1 Victoria Buildings, Weston-super-Mare. |
| *Rudge, C. King | 145 White Ladies Road, Bristol. |
| Russell, M. W. H. | Royal United Hospital, Bath. |
| Salmon, W. G. | Thornbury. |
| Sanctuary, Thomas, M.D. Ed. | Crane Lodge, Salisbury. |
| Scott, James, M.B., C.M. Ed. | 7 St. Mary's Vale, Chatham. |
| Scott, Richard J. H. | 13 Bladud Buildings, Bath. |
| Searle, George C. | Brixham. |
| Seward, W. J., M.B. Lond. | County Asylum, Colney Hatch, Lond., N. |
| Sewart, J. H. | Lostwithiel. |
| Shapter, Lewis, M.D. Cantab. | The Barnfield, Exeter. |
| *Shaw, J. E., M.B., C.M. Ed. | 11 Lansdown Place, Clifton. |
| Sheen, A., M.D. St. And. | 23 Newport Road, Cardiff. |
| Sheldon, T. Steele, M.B. Lond. | Somerset County Asylum, Wells. |
| *Shorland, Walter E. | 211 Cheltenham Road, Bristol. |
| Skeate, Edwin | 16 Paragon, Bath. |
| *Skelton, Henry | Downend. |
| *Skerritt, E. Markham, M.D., B.S., B.A. Lond. | Richmond Hill, Clifton. |
| *Skinner, Stephen, M.B., C.M. Aberd. | Ferndale, Clevedon. |
| Smith, George | Axbridge. |
| *Smith, George | The Coombe, Westbury-upon-Trym. |
| *Smith, G. Munro | 70 Pembroke Road, Clifton. |
| *Smith, J. Greig, M.B., C.M., M.A. Ab., F.R.S.E. | 16 Victoria Square, Clifton. |
| Smith, J. Sidney, M.D. St. And. | Tiverton. |
| *Smith, R. Shingleton, M.D., B.Sc. Lond. | 9 Richmond Hill, Clifton. |
| Smith, Samuel | 7 Kingsdown Parade, Bristol. |
| Society, Cardiff Medical (*Hon. Sec.*, T. Garrett Horder.) | |
| Society, Plymouth Medical (*Hon. Sec.* Dr. A. H. Bampton). | |
| Society, Royal Medical and Chirurgical | 53 Berners Street, London, W. |
| Somer, James | Broadclyst. |
| *Spencer, W. H., M.D., M.A. Cantab. | 5 Lansdown Place, Clifton. |
| Spender, John K., M.D. Lond. | 17 Circus, Bath. |

| | |
|---|---|
| Stansfeld, G. M. | 19 Victoria Square, Clifton. |
| Steel, S. H., M.B. Lond. | Abergavenny. |
| *Steele, Charles, M.D. Dur. | 17 Richmond Hill, Clifton. |
| *Steven, Alexander, M.D., C.M. Ed. | 6 West Mall, Clifton. |
| *Stevens, Frederick G. | 180 Stapleton Road, Bristol. |
| *Stewart, James, B.A. Qu. Univ. I. | Sneyd Park, Bristol. |
| Stock, G. | Barton Hill Road, Bristol. |
| Stockwell, Fred., M.D. Lond. | Bruton. |
| Storry, Frederick W. | Stroud. |
| *Swayne, J. G., M.D. Lond. | 74 Pembroke Road, Clifton. |
| *Swayne, S. H. | 129 Pembroke Road, Clifton. |
| Sydney-Turner, A. M. | College Green, Gloucester. |
| Tayler, Geo. Christopher, M.D. Lond. | Trowbridge. |
| Taylor, Frank | 5 Colston's Parade, Bristol. |
| *Taylor, J. | 5 Ashley Road, Bristol. |
| Taylor, Thomas Henry | Prospect House, Thornbury. |
| Terry, Henry G. | 16 Green Park, Bath. |
| Thomas, A. Garrod, M.D., C.M. Ed. | 28 Bridge Street, Newport, Mon. |
| Thomas, R. W. | Tregare House, Keynsham. |
| *Thompson, George, M.D. Dur. | City and County Asylum, Stapleton. |
| Thomson, G. J. C., M.B. Dur. | Rook Lane House, Frome. |
| Thomson, G. S., M.D. Ed. | 8 College Road, Clifton. |
| Thomson, Spencer, M.D. St. And. | Ashton, Devon. |
| *Tivy, W. J. | 8 Lansdown Place, Clifton. |
| Todd, W. J. | North Petherton. |
| Tosswill, Louis H., M.B. Cantab. | 49 Magdalen Street, Exeter. |
| Twining, A. H. | The Knoll, Salcombe. |
| Tyley, R. P., M.D. St. And. | Wedmore. |
| Valentine, Edmund W. | Somerton. |
| Wade, A. Law, M.D. Dub. | Somerset County Asylum, Wells. |
| *Waldo, Henry, M.D., C.M. Aberd. | 19 Pembroke Road, Clifton. |
| Wallace, Rev. C. Hill, M.A. Oxon. | 3 Harley Place, Clifton. |
| Walsh, David H. | Sneyd Park, Bristol. |
| Walter, William Henry | Knapp Villa, South Petherton. |
| Ward, J. L. W. | Victoria Street, Merthyr Tydvil. |
| *Wathen, J. Hancocke | 18 Richmond Hill, Clifton. |
| Watson, J. Adam | Chudleigh. |
| Watters, George T. B., M.D., C.M. Ed. | Stonehouse. |
| Waugh, Alexander | Midsomer Norton. |
| *Weatherly, Lionel A., M.D., C.M. Aberd. | Portishead. |
| Webb, W. H. | Kingsbridge. |
| *Webster, Thomas | Redland Hill, Bristol. |
| *Welsh, F. F. | Rockleaze Point, Stoke Bishop. |
| Whitfeld, W. Clarke | 5 St. Ethelbert Street, Hereford. |
| Wickham, W. | Hill House, Tetbury. |
| Wicksteed, Francis W. S. | Chester House, Weston-super-Mare |
| Willcox, R. Lewis | Warminster. |
| Willett, M., M.D. St. And. | 87 Ashley Road, Bristol. |
| Williams, Eubulus, M.D. St. And. | 1 Lansdown Place, Clifton. |
| Williams, William Henry, M.D. Lond. | Sherborne. |
| Wilton, John P. | 10 College Green, Gloucester. |
| Winterbotham, L. | Bays Hill, Cheltenham. |
| Wise, N. V. | Fore Street, Trowbridge. |
| Woodd, H. T. | Calstock. |
| Workman, C. J., M.D. Ed. | Teignmouth. |

NOTES ON BOOKS ... ... 63

MEDICAL EXTRACTS ... ... 70

### BRISTOL MEDICO-CHIRURGICAL SOCIETY.

Medical Men wishing to join the Society are requested to communicate with the Honorary Secretary, Dr. R. SHINGLETON SMITH, 9 Richmond Hill, Clifton. The Annual Subscription is 10/6.

*Extracts from Journal By-laws.*

4.—The *Journal* shall be delivered free to Subscribers and also to Members of the Society whose subscriptions are not in arrear.

6.—The Annual Subscription to the *Journal* for those who pay before the 1st of July shall be 5/-, post free, or 6/- if paid after that date.

Communications intended for insertion in the *Journal*, Books for Review, &c., should be sent to the Editor, J. GREIG SMITH, M.A., F.R.S.E., 16 Victoria Square, Clifton.

Exchange Journals and communications referring to the delivery of the *Journal* should be sent to the Publication Secretary, L. M. GRIFFITHS, 9 Gordon Road, Clifton, to whom Subscriptions are to be paid *in advance.*

Authors requiring Reprints of their Papers should communicate with the Publisher.

